# 2377 Evolution of the interdisciplinary co-citation network supported by the Georgia Clinical and Translational Science Alliance Program from 2006 through 2016

**DOI:** 10.1017/cts.2018.283

**Published:** 2018-11-21

**Authors:** Dorothy R. Carter, Nicole Llewellyn, Eric Nehl, Latrice Rollins

**Affiliations:** 1 The University of Georgia; 2 Emory University; 3 Moorehouse School of Medicine

## Abstract

OBJECTIVES/SPECIFIC AIMS: The National Institutes of Health (NIH) has provided continual support for the Georgia Clinical and Translational Science Alliance (CTSA) since 2006. An overarching goal of the Georgia CTSA is to accelerate clinical and translational research to impact health in Georgia and beyond. Toward these ends, a primary objective has been to support interdisciplinary research projects encompassing 2 or more disciplinary domains. The goal of the present study is to evaluate the degree to which interdisciplinary research projects increased in prevalence during the first decade of funding. METHODS/STUDY POPULATION: We began by using PubMed to identify all publications citing the Georgia CTSA hub (n=1865), categorizing each article as encompassing 1 or more research domain using a taxonomy derived from the Web of Science. We created 1 network for each of the 10 years with nodes representing research areas and ties between pairs of nodes representing the presence of 1 or more publication integrating both research areas. We conducted longitudinal network analyses using an approach called MCMC MLE Temporal Exponential Random Graph Models, which models the antecedents of networks over time. RESULTS/ANTICIPATED RESULTS: Supporting Georgia CTSA objectives, results suggest the probability of publications connecting multiple research areas increased over time, with substantially greater increases appearing initially as compared to later years. DISCUSSION/SIGNIFICANCE OF IMPACT: This study advances an innovative approach to modeling the system-wide impact of CTSA hub funding.

